# Artificial intelligence (AI) in psychotherapy: A challenging frontier

**DOI:** 10.1177/10398562251346075

**Published:** 2025-05-27

**Authors:** Daniel Jesudason, Stephen Bacchi, Tarun Bastiampillai

**Affiliations:** Faculty of Health & Medical Sciences, 1066 The University of Adelaide, Adelaide, SA, Australia; Faculty of Health & Medical Sciences, 1066 The University of Adelaide, Adelaide, SA, Australia; Department of Neurology, 1811 Harvard Medical School, Boston, MA, USA; Department of Neurology, 2348 Massachusetts General Hospital, Boston, MA, USA; College of Medicine & Public Health, 1065 Flinders University, Bedford Park, SA, Australia; Department of Psychiatry, 2541 Monash University, Clayton, VIC, Australia; Division of Mental Health, Flinders Medical Centre, Bedford Park, SA, Australia

**Keywords:** machine learning, artificial intelligence, psychotherapy, psychiatry, neurology

## Abstract

**Objective:**

Artificial intelligence (AI) chatbots have emerged as a potential tool to revolutionise mental health care, offering innovative solutions for the diagnosis and management of psychiatric conditions. AI psychotherapy is being trialled as a possible replacement or adjunct to traditional human-led therapy, showing promise in enhancing the accessibility and personalisation of mental health care. This paper seeks to explore the potential risks of AI for psychotherapy.

**Conclusions:**

AI psychotherapy represents relatively unchartered territory. There are concerns surrounding the trainability of AI chatbots, as well as the ultimate ability for an AI to effectively deliver human-like care. We must also consider other consequences, such as the potential for technological misuse. Thus, as AI continues to evolve, we must approach its integration with caution, and ensure the necessary regulatory mechanisms are in place for its effective and equitable implementation.

Psychiatric disorders are a leading cause of morbidity worldwide, contributing significantly to the global burden of disease.^1^ Despite some advancements in care, the number of individuals affected continues to grow, particularly in low-resource settings where challenges in psychiatric management are more pronounced.^2^ This underscores a critical global need for innovative and accessible solutions to improve the diagnosis and treatment of these high-prevalence conditions. In recent years, artificial intelligence (AI) has emerged as a leading technology, offering novel approaches to the diagnostic and management aspects of many disease processes.^1,3^

AI is defined as any algorithm or computational system capable of performing functions such as learning, reasoning, pattern recognition, and decision-making, often through techniques such as machine learning, deep learning, neural networks, and natural language processing.^1^ The uses of AI systems in psychiatry have been widespread, and can be broadly categorised into four domains, as shown in Figure 1. AI has primarily been utilised in the diagnosis, monitoring, and management of mood and anxiety disorders.^1,3,4^

**Figure 1.**
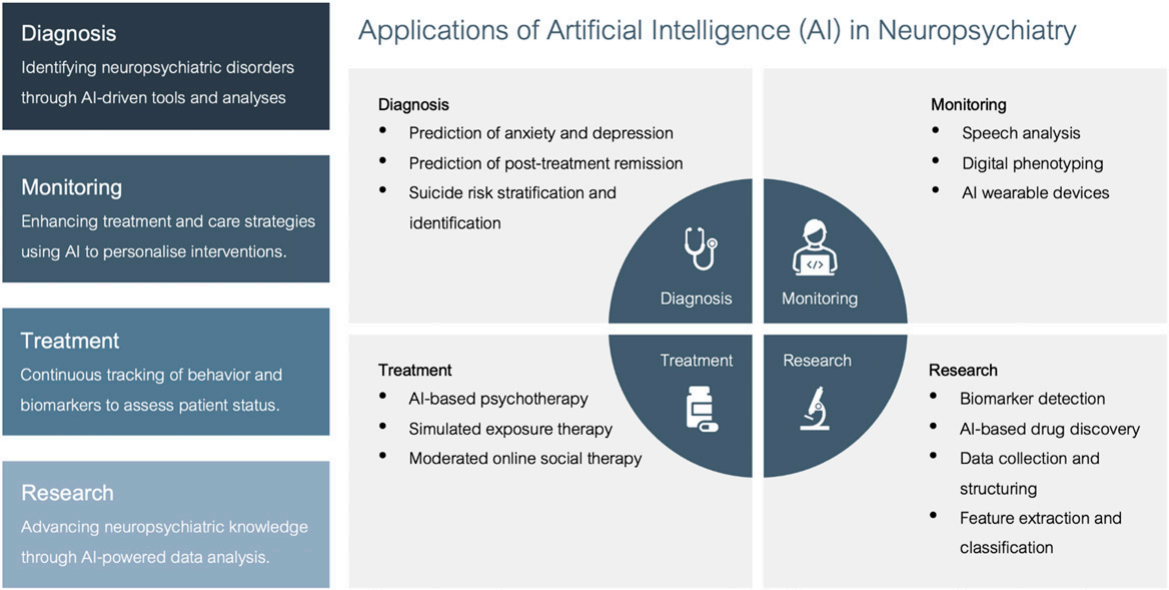
An infographic depicting the various application of AI in psychiatry.

AI psychotherapy describes the use of AI systems to provide psychological services equivalent to those offered by a human therapist.^1^ This treatment modality was conceived because of its potential to provide mental health care in an accessible and cost-effective way, thus providing benefit to individuals in low-income and low-resource demographics, with limited access to mental healthcare professionals.^5^ The majority of AI psychotherapy services are delivered through AI chatbots, which are computer programs designed to simulate human conversation. Chatbots perform functions such as providing emotional support during psychological distress, and guiding users through therapeutic interventions like cognitive-behavioural therapy (CBT). They are trained using large datasets of human language, enabling them to respond adaptively to users’ requests. However, AI psychotherapy is yet to receive large-scale implementation and has only been tested in limited models (see Table 1). A few studies have demonstrated the ability of AI systems to provide care comparable to a traditional provider.^1,3,6^

**Table 1.** A table summarising some common services that deliver AI psychotherapy.

Service	Developer	Services offered
*Woebot*	Woebot health	Conversations; CBT; healthy lifestyle support
*Wysa*	Touchkin eServices	Conversations; CBT
*ElizaGPT*	OpenAI	Conversations; CBT; guided self-exploration
*Ebb*	Headspace	Conversations; healthy lifestyle support
*Tessa*	NEDA	Conversations; weight loss support

These preliminary studies have sparked significant interest in the development and refinement of AI psychotherapy.^7^ In this perspective, given the global revolutionary impact of AI in almost all industries, it is important to specifically evaluate the risks of implementing AI for psychotherapy.

## Challenges of AI psychotherapy

### Algorithmic biases and training

One significant criticism of AI psychotherapy is its inability to understand and adapt to diverse sociocultural contexts in a manner similar to a human therapist.^3^ Most AI algorithms are programmed using Reinforcement Learning from Human Feedback (RLHF), a training method where models learn to optimise their behaviour based on feedback from humans. However, this means that the performance of an AI is almost solely based on its algorithmic programming.^8,9^ For example, most AI algorithms are trained primarily on male demographic data, resulting in a lack of sensitivity to issues that women face, leading to the minimisation of experiences and even the perpetuation of maladaptive stereotypes.^10^ Superficially, this seems like a trivial issue that could easily be resolved. However, when extending algorithmic biases to metrics such as race, culture, political affiliation, age, socioeconomic standing, and cognitive ability, it becomes increasingly more difficult to create a sufficiently representative model.

A cogent counter-argument to this is that the same case could be made for a human; it is almost equally as impossible to train a human to truly empathise with the lived experiences of another if they have not shared similar experiences themselves. While true to an extent, this argument fails to acknowledge that, although a human therapist may not have personally experienced the particular circumstances of their clients, they would likely have felt analogous emotions as an adherent to the traditional human experience. Furthermore, humans can demonstrate iterative adaptive learning to a much greater extent than AI and are thus capable of utilising past clinical encounters to shape their future interactions. An equivalent capability in an AI psychotherapy setting would be almost impossible to achieve without raising significant concerns regarding the confidentiality and ethical handling of patient data. Finally, human therapists can engage in introspection and metacognition, and thus consciously identify and address their own biases in real-time, in a way that AI currently cannot.

Moreover, conventional AI chatbots, such as GPT-4 (ChatGPT) and Gemini, are trained from datasets that reflect the needs of the general population, rather than psychiatric patient cohorts. As such, most existing AI psychotherapy systems are not recommended for clinical purposes and are instead intended to serve as demonstrative or educational tools. One tool that was intended for clinical use was *Tessa*, the AI chatbot aimed at providing therapeutic services to patients with eating disorders. However, *Tessa* was infamously redacted for providing ‘harmful advice’, after only 1 week of use. The fallout from *Tessa*’s pilot suggests that more robust evidence must be collected before AI chatbots are implemented in a clinical setting.^11^

### Wisdom and empathy

Even with superior training that better represents the parameters of psychiatric patient cohorts, it is unlikely that an AI therapist could truly replicate the experience provided by a human therapist. AI systems are undoubtedly ‘intelligent’; they have proven to be far superior to humans in many facets of intellect, including quantitative reasoning, auditory processing, visuospatial ability, and data comprehension.^12^ The term ‘wisdom’ has surfaced as a more appropriate description of what human intellect entails, and what AI models should aim to replicate within the clinical space.^13^ Wisdom is a multi-dimensional personality trait that encompasses behaviours such as empathy, compassion, emotional regulation, and self-reflection, which are almost impossible to fully emulate in a non-human entity. Wisdom, rather than intelligence, is what patients value within their therapist; they want the physical presence of a compassionate individual that establishes a unique sense of connection and security.^14^

Similarly, AI psychotherapy models have demonstrated an inability to deliver criticism, which is often a critical component of psychotherapy. By design, RLHF deliberately trains AI models to prioritise unconditional positive regard, in order to optimise user satisfaction.^15^ Thus, it is often difficult for an AI model to engage processes that facilitate adaptive change, such as cognitive restructuring, as they are hardwired to create short-term user satisfaction, often at the expense of longer-term benefits. If an AI cannot reconcile its patient with the reality of their situation, offer criticism, and promote adaptive change, then its utility as a therapeutic adjunct comes into question.

### Iatrogenic dependency

It is also possible that AI psychotherapy could be utilised by patients to a greater extent than traditional therapy, given the shortages of healthcare providers and historically long waitlists to see mental health professionals. Having access to continuous and on-demand care is very much unprecedented in the healthcare sector and introduces concerns surrounding the potential for patient dependency upon AI systems. For example, a patient with an anxiety disorder may find comfort in having a constant source of unconditional positive regard and reassurance. As such, the AI may inadvertently enable a patient’s validation-seeking tendencies, reducing their capacity to self-regulate and coexist with the inevitable uncertainty of life. Thus, providing an unlimited source of unconditional positive regard could ultimately dysregulate an individual’s sense of self, leading to issues with self-esteem, self-perception, and mood.

### Ethical implications

The ethical implications of AI psychotherapy have been well described in the literature and are largely representative of the broader concerns with the use of AI in general. Concerns regarding data privacy, confidentiality, liability, and sustainability have been raised, and will therefore need to be addressed by governmental systems and electronic medical record (EMR) services if mainstream implementation is achieved.^16-18^

A dystopian job takeover is commonly depicted as another implication of using AI in medicine. However, current evidence suggests that AI psychotherapy will most likely not reach the point of being used as a standalone source of psychiatric intervention and will instead likely serve as simply one component of an integrative mental health care ecosystem.^16,18^ This ecosystem will incorporate various technological modalities, and feature services such as digital sleep tracking, automated exercise tracking, and AI-guided meditation, in addition to in-person psychiatric care. Thus, AI psychotherapy should not be considered as a panacea that replaces the purpose of a human therapist, but rather as one component of a broader management plan.

Another ethical challenge pertains to the provision of equitable healthcare. A commonly cited benefit of AI psychotherapy is its potential to serve low-income demographics, by offering a cost-effective psychiatric treatment modality.^19,20^ However, this introduces the potential for an oversaturated online market, with various products that drastically differ in price and quality. Furthermore, a study by Fiske et al. (2019) suggests that there are even concerns that an AI psychotherapy market would justify the closure of existing psychotherapy services, thus exacerbating existing health inequity in low-access areas.^21^ Thus, AI psychotherapy services must be held accountable and governed by strict oversight, to prevent market oversaturation, maintain clinical rigour, and ensure the permanency of traditional services.^20^

## Conclusion

The use of AI chatbots in psychotherapy represents an opportunity to revolutionise the care of patients living with anxiety and mood disorders. However, although AI psychotherapy offers the unprecedented opportunity to improve access, efficiency, and innovation in mental health care, its potential downstream consequences raise important questions about its eventual place in the modern therapeutic process. As we navigate this ever-evolving landscape, we must remain mindful of the intangible human factors central to therapy, including wisdom, trust, empathy and attachment. Ultimately, the long-term effects of AI psychotherapy are largely uncharted, offering an opportunity for ongoing research into the implications of this technology. Thus, the challenge lies not only in refining these AI technologies but also in reimagining how they can ethically and effectively coexist with traditional therapy approaches.
